# Supplementary material for: Dairying enabled Early Bronze Age Yamnaya steppe expansions
Source: Nature. 2021 Sep 15;598(7882):629–33. doi: 10.1038/s41586-021-03798-4 (PMC8550948; doi:10.1038/s41586-021-03798-4)
Supplement: Supplementary file 1 — This file contains supplementary text, supplementary figures s1 – s2 and supplementary references. [file 41586_2021_3798_MOESM1_ESM.pdf]

nature portfolio

https://doi.org/10.1038/s41586-021-03798-4

Supplementary information

Dairying enabled Early Bronze Age Yamnaya steppe expansions

In the format provided by the authors and unedited

Dairying enabled Early Bronze Age Yamnaya steppe expansions

Supplementary Information

Shevan Wilkin[1,2*], Alicia Ventresca Miller[1,3], Ricardo Fernandes[1,4,5], Robert Spengler[1], William T.T. Taylor[1,6], Dorcas R. Brown[7], David Reich[8,9,10,11], Douglas Kennett[12], Brendan J. Culleton[13], Laura Kunz[14], Claudia Fortes[14], Aleksandra Kitova[15], Pavel Kuznetsov[16], Andrey Epimakhov[17,18], Victor F. Zaibert[19], Alan K. Outram[20], Egor Kitov[21,22], Aleksandr Khokhlov[16], David Anthony[7,11], Nicole Boivin[1,23, 24, 25*]
*Corresponding authors

1. Department of Archaeology, Max Planck Institute for the Science of Human History, Jena, 07745, Germany

2. Institute for Evolutionary Medicine, Faculty of Medicine, University of Zürich, Zürich 8057, Switzerland

3. Department of Anthropology, University of Michigan, Ann Arbor, Michigan, 48109, USA

4. School of Archaeology, University of Oxford, South Parks Road, Oxford OX1 3TG, Oxford, United Kingdom

5. Faculty of Arts, Masaryk University, Arne Nováka 1, 602 00 Brno-střed, Czech Republic

6. Department of Anthropology, University of Colorado, Museum of Natural History, Boulder, CO, 80309, USA

7. Department of Anthropology, Hartwick College, Oneonta, NY 13820, USA

8. Department of Genetics, Harvard Medical School, Boston, MA 02115, USA

9. Broad Institute of Harvard and MIT, Cambridge, MA 02142, USA

10. Howard Hughes Medical Institute, Harvard Medical School, Boston, MA 02115, USA

11. Department of Human Evolutionary Biology, Harvard University, Cambridge, MA 02138, USA

12. Department of Anthropology, University of California, Santa Barbara, CA 93106 USA

13. Institutes of Energy and the Environment, The Pennsylvania State University, University Park, PA, 16802 USA

14. Functional Genomics Centre Zürich, University of Zürich/ETH, Zürich 8057, Switzerland

15. Center for Egyptological Studies, Russian Academy of Sciences, Moscow 119071, Russian Federation

16. Samara State University of Social Sciences and Education, Samara, Russian Federation

17. South Ural State University, Chelyabinsk 454080, Russia

18. Institute of History and Archaeology, Ural Branch RAS, Yekaterinburg 620990, Russia

19. Institute of Archaeology and Steppe Civilizations, Al-Farabi Kazakh National University, 71 Al-Farabi St., Almaty, Kazakhstan

20. Department of Archaeology, University of Exeter, Exeter EX4 4QE, UK

21. Center of Human Ecology, Institute of Ethnology and Anthropology, Russian Academy of Sciences, Moscow 119991, Russia

22. Faculty of History, Archaeology, and Ethnology, Al-Farabi Kazakh National University, Almaty 050040, Kazakhstan

23. School of Social Science, The University of Queensland, Brisbane, QLD 4072, Australia

24. Department of Anthropology and Archaeology, University of Calgary, Calgary, AB, T2N 1N4, Canada

25. Department of Anthropology, National Museum of Natural History, Smithsonian Institution, Washington, D. C. USA

Correspondence to Shevan Wilkin and Nicole Boivin
Email: wilkin@shh.mpg.de; boivin@shh.mpg.de

1. Archaeological information for sites and individuals included in this study

We selected samples for this study from sites and individuals spanning the Eneolithic through to the Late Bronze Age in the Pontic Caspian region. Individuals were sampled from three different institutions: All individuals except those from Kammenyi Ambar 5 and Botai are curated at Samara State University's Department of Archaeology. The three individuals from the site of Kammenyi Ambar 5 belong to the scientific collections of the Museum at the Institute of Plant and Animal Ecology (Ural Branch of the Russian Academy of Sciences). The two samples collected from individuals from the site of Botai were sampled by Alan Outram and delivered to MPI-SHH, the individuals are stored by Victor F. Zaibert at the Botai Research Station. Sampled individuals were dated using both established archaeological material culture associations and radiocarbon dating. While many of the radiocarbon dates had been previously published, we also obtained unpublished new dates issued by the PSUAMD lab at Penn State University through the cooperation of the David Reich Laboratory at Harvard University. We present all cultural assignments and absolute dates here.

We also provide archaeological information on the mortuary sites where the samples were collected for this study; in each case we include site name, date(s), number of individuals, and GPS coordinates; see summary in Supplementary Table S1. All individuals included in this study are adults, and the protein identifications for each are included in Supplementary Table S3. We classified the sites following three date ranges: 1) Eneolithic (*ca*. 4600 to 3300 BCE): 2) Early Bronze Age (*ca*. 3300 to 2500 BCE; EBA); and 3) Middle/Late Bronze Age (*ca*. 2500 to 1700 BCE; MLBA). Dates obtained from direct radiocarbon analysis of human skeletal remains are listed as cal BCE, and those estimated from either archaeological artifact seriation or direct dates on associated material are listed as *ca*. BCE.

Eneolithic sites

During the Eneolithic *(ca*. 4500 to 3300 BCE), the area that today covers western Russia was occupied primarily by sedentary hunter-gatherer-fisher (HGF) populations. Archaeological remains of domesticated ruminants appear at low prevalence in occasional burials, but played an uneven role in the diet, varying from site to site. Stable isotope analysis was used to establish that the typical Eneolithic diet included significant amounts of freshwater fish[1]. Freshwater foods are typically depleted in ^{14}C content when compared to the contemporaneous atmosphere, and their consumption, accordingly, results in radiocarbon dates that appear older, an effect known as the dietary freshwater radiocarbon reservoir effect[2,3]. We employed a Bayesian mixing model and estimates of local freshwater radiocarbon reservoir effects to produce a more accurate age estimate for each Eneolithic individual (the method is described in two papers by Fernandes et al.[2,3] and our main text methods section). Such age estimates result in wider chronological ranges than direct calibration of human radiocarbon measurements and show that actual chronologies of human remains may be younger by several centuries. However, in every Eneolithic sample in the present study, these are still within chronological ranges that do not alter their cultural assignment.

Ekaterinovsky Mys
(Number of Individuals: 1; Individual Archaeology Code: EKAT N-74)

This Eneolithic non-kurgan cemetery contains more than 100 burials and is located in the southern part of the Middle Volga region near the modern city of Samara. Grave goods included items similar to those at Khvalynsk (described below) and domesticated ruminant bones, but, unlike Khvalynsk, the

graves lacked copper items. For example, one male burial from grave number 45 contained the remains of a young goat, three stone maces, and a carved antler in the shape of a bird's head. Traces of ochre staining were apparent above the body along with discarded pottery, representing mortuary rituals. This individual was not the individual sampled for our study, but rather a contemporaneous one. The ceramics in the graves at Ekaterinovka Mys were of a local *northern* style, called the *Samara* type, which is different from Khvalynsk, but similar to other Eneolithic sites along the Samara River. The human remains were extended in the standard burial posture for the Samara region at this time (supine extended), which was different from the Khvalynsk posture (supine and contracted, with raised knees). A domesticated goat tooth from the above-mentioned young goat in grave 45 yielded a date of 4578-4451 calBCE (5680±20 BP/PSUAMS-4568), and four other unpublished dates on terrestrial animals or organic residues fall in the 4700-4500 BCE range, so this cemetery was in use one or two centuries before Khvalynsk. The possibility that Ekaterinovka Mys is slightly older was supported by the near lack of copper artifacts, whereas there are abundant copper finds at Khvalynsk. The human skeleton from grave 45, a male aged 20-25, yielded a radiocarbon date of 5363-5224 calBCE (6325±25 BP, PSUAMS-2880) which was 800 ^{14}C years older than his associated goat offering. Dates on human remains in this cemetery are clearly skewed by the freshwater reservoir effects, like the sampled individual that we used in our study, originating from grave 74[4,5].

Radiocarbon date from the individual sampled in the present study:
EKAT N-74: 5361 to 4555* cal BCE (6330±25 BP, PSUAMS-4313)[4]
*The calibrated date for this individual is the modeled date for freshwater radiocarbon reservoir effect.

Khlopkovsky Bugor
(Number of Individuals: 2; Individual Archaeology Codes: KB N-7; KN N-8)

This Eneolithic flat cemetery contains 24 burials and is located in the Middle Volga region on the right bank of the river. The associated artifacts have been assigned to the Khvalynsk archaeological culture. Individuals from the site fall on the same genetic cline as those at Khvalynsk, and our sampled individual at Khlopkov Bugor, the female in grave 7, was a second-degree relative (possibly an aunt or grand-mother) of a male buried at Khvalynsk II[6].

Radiocarbon date from the individual sampled in the present study:
KB N-7: 5211 to 4339 cal BCE* (6170±25 BP, PSUAMS-2908[7]).
*The calibrated date for this individual is the modeled date for freshwater radiocarbon reservoir effect.

Khvalynsk I
(Number of Individuals: 5; Individual Archaeology Codes: KHA1 N-A; KHA1 N-39, KHA1 N-61/62 KHA1 N-125; KHA1 N-127)
Khvalynsk II
(Number of Individuals: 4; Individual Archaeology Codes: KHA2 N-1, KHA2 N-2; KHA2 N-12; KHA2 N-4)

These two cemeteries are situated in the Middle Volga region on the right bank of the river. Excavators recovered 158 individuals from Khvalynsk I, while Khvalynsk II contained 39 graves with 43 individuals. These two cemeteries were 120 m apart and exhibit the same dates and archaeological culture, and were almost certainly used by the same population. Khvalynsk I presents a normal demographic profile of adult males, females, and children, but Khvalynsk II contained 3 males for

each female and few children, and seems to have been a burial place primarily for adult males with greater abundances of copper offerings. The Khvalynsk I and II cemeteries collectively contained more than 370 copper items, probably made of imported Balkan copper, the largest number of copper objects from any fifth-millennium BCE site in the Pontic-Caspian steppes. Archaeological and isotopic data suggest that people maintained a diet focused on fish and wild resources, as well as a smaller percentage of domesticated animals. The human remains generally date between 400-700 years older than the terrestrial animals included in the same graves, indicating a reservoir effect from the consumption of aquatic resources (See Supplementary Table S4). Sheep and cattle bones from the site have been dated to between *ca.* 4500 to 4300 BCE. While there are a small number of clearly domesticated animals in these burials, archaeologists have assumed that these animals were used in more of a ritual manner rather than as a consistent feature of daily subsistence[6,8,9].

A date of 4450-4355 cal BCE was obtained on a ring made of sheep bone (GrA-29178, 5565±40 BP) from grave 147 at Khvalynsk I. The human female buried with this bone ring was dated to 4789-4618 cal BCE (PSUAMS 2886, 5845±25 BP); this suggests that there was a 280±147 ^{14}C year discrepancy, due to the reservoir effect. A date of 4448-4362 cal BCE (GrA-34100, 5570± 40 BP) was obtained on a cow bone from grave 10 at Khvalynsk II. These ruminant grazers would not have been subject to the reservoir effect, with dates of 4450–4355 cal BCE from Khvalynsk I and 4448–4362 cal BCE from Khvalynsk II. These two dates provide the best estimate currently available for the true age of the two cemeteries.

If we compare the radiocarbon ages of the ruminants to those of the humans, the resulting offsets range between 55±47 and 405±47 ^{14}C years.

Radiocarbon dates from individuals sampled for the present study:
KHA2 N-127: 4499 to 3800 cal BCE* (5625±25 BP, PSUAMS-2885)
KHA2 N-1: 4685 to 3818 cal BCE* (5760±25 BP, PSUAMS-4032)
KHA2 N-4: 4899 to 4056 cal BCE* (5965±20 BP, PSUAMS-2903)
KHA2 N-2: 4932 to 4058 cal BCE* (5975±25 BP, PSUAMS-2902)
KHA2 N-12: 4900 to 4059 cal BCE* (5960±25 BP, PSUAMS-4031)
*The calibrated date for this individual is a modeled date for freshwater radiocarbon reservoir effect (See Supplementary Table S5).

Murziha II
(Number of Individuals: 6; Individual Archaeology Codes: MUR2 N-98; MUR2 N-94/7; MUR2 N-128; MUR2 N-130.1; MUR2 K-1 N-130; MUR2 N-91-1)

The multi-period burial ground (n=19 burials) is located in the northern forest region of the Volga-Kama River. The Eneolithic burials are related to the Ust-Kama culture, and funeral rituals appear to share some similarities to those from Ekaterinovsky Mys. While the Khvalynsk burials also contained domesticated animal bones, the Murzikha II burials did not. However, at a similar and nearby cemetery, Gulkinsky II, a *Bos* sp. scapula (probably a tool) was included in grave number 124, and some bones of domesticated calves were also recovered from the grave cluster. Several graves at Murzikha II contained small rings made of copper, a cultural feature also present at Khvalynsk. Ten burials from the site have been dated, including three that are included in this study. The calibrated dates range from 4599 to 3526 cal BCE. The isotopic composition of the bones has not been studied, but the associated artifacts indicate that the diet was likely hunting and fishing-based; hence, we can assume that the reservoir effect has influenced the dates[10].

Radiocarbon dates from individuals sampled in the present study:
MUR2 N-91-1: 4552 to 4340 cal BCE (5610±60 BP, GIN-9436)
MUR2 N-94: 4541 to 4361 cal BCE (5630±40 BP, GIN-9428)
MUR2 N-128: 4364 to 4052 cal BCE (5390±60 BP, GIN-10039)

Early Bronze Age Yamnaya (Pit-Grave) sites

The earliest radiocarbon dates associated with graves that have artifacts assigned to the Yamnaya culture fall between *ca*. 3300-3000 BCE. The Yamnaya culture is normally equated with the beginning of the EBA in the chronology of the Pontic Caspian region. This region is seen as a focal one where a number of key genetic and cultural traits critical to the development of the Yamnaya were assembled, with potential links to the oldest phase of Proto-Indo-European[6]. The Yamnaya culture introduced marked changes in funeral rites and settlement patterns, and stable isotopes from human bones suggest dietary changes as well. We can now confirm the dietary shift towards a complementary pastoralist component to the existing hunting and fishing economy with new protein evidence for dairy consumption from dental calculus, as presented in this paper. The shift from riverine (Eneolithic) to grassland and riverbank resources (EBA), seen isotopically, was accompanied by the widespread abandonment of riverine Eneolithic residential sites and by the initial appearance of kurgan cemeteries in the grassland plateaus of major river valleys. While zooarchaeological remains are limited, they nonetheless support the evidence for a shift to pastoral resources. Early Bronze Age and Yamnaya zooarchaeological remains in the Volga-Ural study area are limited to occasional sacrifices in graves, 70-90% of which were sheep-goat[52]. In the Dnieper Valley, two Yamnaya settlements had primarily cattle, followed by sheep-goat and horse[45, 48, 49]. Furthermore, a majority of the sites from which the individuals in the present study derive fall across a landscape that would have been ideal for large ruminant herds[11], and archaeologists have suggested that regional Bronze Age populations relied on semi-sedentary cattle, sheep, and goat pastoralism[12–15].

From this data as well as the modeled Bayesian radiocarbon age estimates discussed in the section above, it is evident that there was a dramatic increase in the use of domesticated ruminant animal products in the Early Bronze Age. In comparison to the Eneolithic period, freshwater fish consumption was lower in the Early Bronze Age; and stable isotope studies on Bronze Age populations from the region show that an increase in nitrogen values in humans corresponded to higher values in local herbivores as well rather than from freshwater fish consumption[1,16]. While we acknowledge that fish may have been consumed at a small scale, it likely did not affect AMS dates from this period to the extent of individuals living during the Eneolithic period. Therefore, radiocarbon date ranges for individuals from the Bronze Age were not adjusted (Supplementary Table S5).

Krasnoholm III
(Number of Individuals: 1; Individual Archaeology Codes: KRA3 K.1 N-1)
This burial mound was discovered on the left bank of the Ural River in the Pre-Urals region. It is one of the three mounds that contained artifacts ranging in age from the EBA to the Early Iron Age. The individual in our study was buried in a grave in the style of the Yamnaya culture. It was a single burial, sprinkled with ochre, and the grave included charcoal and four polished bone tubes. The complex has not yet been radiocarbon dated[17].

Krasikovskyi I
(Number of Individuals: 2; Individual Archaeology Code: KRS K.2 N-1; KRS K.1 N-1)
This burial mound cluster is located in the Volga-Ural region in the forest-steppe zone of the Tok River, a tributary of the Samara River. The cluster consists of five kurgans, which contained five Yamnaya culture graves (EBA) and one grave associated with the Abashevo culture (MLBA). Early graves with individual burials were disturbed, but accompanying artefacts included a pectoral ornament made of a wild boar tusk and the stone tip of a dart[18].

Radiocarbon dates from the individual sampled for the present study:
(This individual was dated repeatedly over multiple studies by different researchers, and we consider the AMS-date in bold to be the most reliable)
KRS K.2 N-1: **2952 to cal BCE (4259±34 BP, AAR-27477)**
3508 to 3020 cal BCE (4542±70BP, SPb-2092)
3488 to 3037 cal BCE (4535±50BP, SPb-2093)
2872 to 2580 cal BCE (4130±40 BP, Ki-19485)
2623 to 2473 cal BCE (4030±30 BP, Ki-19480)
3340 to 2913 cal BCE (4425±67 BP, IMCES 14C-1521).

Krivyanskyi IX
(Number of Individuals: 2; Individual Archaeology Codes: EBA: KRI9 K.4 N-21; KRI9 K.2 N-2)
This burial ground is located between three small rivers in the Don basin, 30 km northeast of Rostov-on-Don. It consists of seven kurgans that in total contained 92 burials. The site was used from the Eneolithic through the LBA, and also included some later household pits of the medieval Khazar period. The analyzed archaeological remains come from four mounds; the earliest phase of mound construction dates to the EBA Yamnaya culture (No. 1, 2, 4) and MBA (No. 5). The EBA burials were in pit graves, the MBA burials were interred in catacombs. Individual and collective burials (up to five skeletons), with traces of ochre, were characteristic of both periods. There were few funeral offerings or artifacts in any of these graves; and most of the deceased had no accompanying artefacts. Exceptions include a single ceramic vessel in one burial from the Yamnaya culture, as well as a stone flake and small cattle bones in one of the MBA Catacomb burials[19].

Radiocarbon dates from individuals sampled in the present study:
KRI9 K.4 N-21A: 3345 to 3096 cal BCE (4495±25 BP, PSUAMS-7979) This was a collective grave containing five individuals, two adults and three immatures. The dental calculus sample was taken from adult A, pictured below. A second date on adult C was significantly different: 2904-2701 calBCE (4225±25 BP, PSUAMS-7980).

KRI9 K.2 N-2: 2881 to 2633 cal BCE (4165±25 BP, PSUAMS-7978) This was a single grave containing an EBA ceramic vessel.

Kutuluk I
(Number of Individuals: 2; Individual Archaeology Codes: KUT1 K.3 N-4; KUT1 K.4 N-1)
This is a kurgan cluster in the Volga-Ural region that consisted of four kurgans, constructed during the EBA. Grave 1 in kurgan 4 is heavily cited, due to the presence of a male with a mace and metal weapons[20]. Two radiocarbon dates have been run on this individual. The other burials likely also belong to the early phase of the Yamnaya culture.

Radiocarbon date from the individual sampled in the present study:
KUT1 K.4 N-1: 3360 to 2928 cal BCE (4470±70 BP, OxA-4306)[21]

Leshchevsky 1
(Number of Individuals: 1; Individual Archaeology Code: LES K.1 N-1)
This burial mound is located in the Middle Volga steppe zone on the Samara River and two of the three kurgans at the site have been investigated. Two central graves contained artifacts linked to the Yamnaya culture and included a metal dagger and stone tools. Our sample was taken from the skeleton of a young woman, and two dates for this individual are presented here.

Radiocarbon date from the individual sampled in the present study:
LES K.1 N-1: 3091 to 2918 cal BCE (4390±20 BP, PSUAMS-4159)

Lopatino I
(Number of Individuals: 1; Individual Archaeology Code: LOP1 K.3P N-1)
Lopatino I is a large kurgan cemetery containing 35 burial mounds located on the Sok River, in the forest-steppe zone on the left bank of the Middle Volga. Eight burial mounds have been excavated, yielding pit graves of the Yamnaya (EBA), Poltavka (MBA), and Srubnaya (LBA) cultures[22].

Radiocarbon date from the Yamnaya individual sampled in the present study:
LOP1 k.3p N-1: 3522 to 3020 cal BCE, (4560±80 BP, Ki-7764)

From a pit associated with this grave:
3339-2916 cal BCE (4432±66 BP, AA-47804)

Mustayevo V
(Number of Individuals: 2; Individual Archaeology Codes: MUS V K.1 N-1; MUS V K.8 N-2)
The site was located on the right bank of the Ural River in the northern steppe Pre-Urals region. The three mounds investigated during excavations contained burials of the Yamnaya and Srubnaya cultures, as well as burials of the Early Iron and Middle Ages. The samples examined in the present study were obtained from the Yamnaya culture burials and the skeletons had been sprinkled with ochre and interred with grave goods including a ceramic vessel[23].

Radiocarbon dates from individuals sampled in the present study:
MUS V K.1 N-1: 2698 to 2488 cal BCE (4070±30 BP, IGAN-2780)
MUS V K.8 N-2: 3377 to 2903 cal BCE (4480±100 BP, IGAN-3016)[23].

Nizhnaya Pavlovka V
(Number of Individuals: 1; Individual Archaeology Code: NP V K.2 N-3)
This burial mound cluster was discovered on the left bank of the Ural River in the steppe Pre-Urals; three mounds were excavated, and the earliest burials contain artifacts associated with the Yamnaya culture. Unusual features from the grave of the individual in this study included a massive metal hammer-axe. Sheep bones accompanied one of the burials.

Radiocarbon date from the individual sampled from a previous study (not from the individual analyzed in the present study):
2886 to 2662 cal BCE. (4175±35BP, GrA-54391)[24].

Panitskoe 6B
(Number of Individuals: 1; Individual Archaeology Code: PAN N-6)
A single kurgan, Panitskoe 6B, was excavated in the steppe zone of the Lower Volga region, along the Karamysh River, a tributary of the Volga. The burial mound contained seven burials, including an EBA grave with two individuals (adult male and child)[25]. The mortuary tradition and grave goods included ochre, vessels of the Khvalynsk-Berezhnovka type, and a bone pin. All of these features have attributed it to the early phase of the Yamnaya complex.

Radiocarbon date from the individual sampled in the present study:
PAN N-6 (adult): 3516 to 2904 cal BCE (4500±120 BP, Ki-13050)
On Yamnaya grave 1 3346-3101 calBCE (4505±20 BP, PSUAMS-4161)

Podlesny
(Number of Individuals: 1; Individual Archaeology Code: POD K.1 N-3)
This mound cluster was discovered on the left bank of the Samara River, a tributary of the Volga in the Middle Volga forest-steppe region, and two out of the eight mounds in the cluster have been excavated. The excavators believed that the burials belonged to the Poltavka and Srubnaya cultures; however, the ceramic vessel that was used to assign a phase to the burial was not found at the bottom of the grave (which contained only the skeleton), but rather higher up. Judging by the AMS date for the individual in our study, the burial belongs to the Yamnaya culture. The location of the skeleton, its orientation, and the use of ochre do not contradict this assumption.

Radiocarbon date from the individual sampled in the present study:
POD K.3 N-3: 3330 to 3028 cal BCE (4465±20 BP, PSUAMS-4412);

Pyatiletka
(Number of Individuals: 1; Individual Archaeology Code: PYA K.6 N-2)
Three mounds were found and excavated on the left bank of the Ural River in the northern Pre-Urals region. The main burials of each barrow belong to the Yamnaya culture, as determined by the specific grave features (ochre, burial position, and inhumation style). Some noteworthy features of these burials include rare examples of paired burials, a ditch around a mound, and metal tools, all of which differentiate this site from other contemporaneous regional sites.

Radiocarbon date from the individual sampled in the present study:
PYA K.6 N-2: 2875 to 2621 cal BCE (4140±35BP, GrA-54392) [17].

Trudovoy II
(Number of Individuals: 1; Individual Archaeology Code: TRU K.5 N-1)
A large burial mound was found on the right bank of the Ural River in the Pre-Urals of the northern steppe. Two of the three largest excavated mounds were erected over the graves of Yamnaya burials. The grave of the individual analyzed in the present study was constructed after the original kurgan monument was completed. It was identified as a Yamnaya burial based on structural details, body position, the presence of a ceramic vessel-censer, and other artifacts. In addition, the mandible of a sheep was found in the burial. The site has no associated radiocarbon dates[24].

Middle and Late Bronze Age Sites

The Middle/Late Bronze Age (*ca*. 2500 to 1700 BCE) of the Pontic Caspian region was dominated by Poltavka, Catacombnaya, Abashevo, Sintashta, Srubnaya, and Andronovo culture groups who relied heavily on horses and ruminant animals[26,27]. Originating from the earlier Yamnaya, these populations shared similar ceramic styles, metal tools, kurgan burials, and chariots[13,20,28]. As opposed to the EBA populations, the Abashevo, Sintashta, Srubnaya and Andronovo groups became more sedentary and began building larger, more permanent settlements[29]. Zooarchaeological evidence combined with stable isotope analysis of Sintashta individuals from western Kazakhstan's Kamennyi Ambar 5 and Bestamak indicate that while diets included both horses and ruminants, they were weighted towards the latter[26,30].

Bolshekaraganskyi
(Number of Individuals: 1; Individual Archaeology Code: BKK Kurgan 25 Grave 24)
This MBA burial cluster has been linked to the Sintashta culture in the Trans-Urals, and continued to be used until the end of the Iron Age. The cemetery contains multiple kurgans (n = 26), some of which have been excavated. At least five are assumed to date to the MBA. Kurgan 25 is a large kurgan with multiple pits, some of them with a single skeleton, while others contained multiple individuals[31,32]. Overall, kurgan 25 contained human and faunal remains, including horse, cow, sheep, and pig bones. From isotopic studies of the humans and domesticated animals, it is clear that humans were consuming animal products, but it is not clear whether they were consuming both meat and dairy[32].

Kalinovsky 1
(Number of Individuals: 2; Individual Archaeology Codes: KAL1 K.1 N-6; KAL1 K.1 N-4)
This burial mound cluster is located in the Middle Volga region on the left bank of Volga River in the forest-steppe zone. It is one of the northernmost sites of the Poltavka culture. Kurgan 1 contained four graves, with artifacts, such as ceramic vessels, a bronze awl, a tool for decorating ceramics, and cattle and sheep/goat bones[33].

Kammenyi Abmar 5
(Number of Individuals: 3; Individual Archaeology Codes: KA5 K.2 MR11 CK-1 A944; KA5 K.2 A987; KA5 K.4 MR-5 CK-2 A937)
The site is well-studied and has been dated to the MBA, specifically between 2040 and 1730 cal BCE. Kammenyi Abmar 5 lies just to the east of the Ural Mountains and close to the Kazakhstan border. Four kurgans (26 graves and more than 100 individuals) contain graves of the Sintashta culture[34–36]. Subsistence at the site is assumed to have been based primarily on pastoralism, but was likely supplemented with wild plants and animals. There is no direct evidence for use of domesticated crops, but the possibility for cultivation cannot be completely discounted. At the site, other material artifacts were also dated to between *ca*. 2000 to 1700 BCE, indicating date ranges closer to the unmodelled human individual dates that did not take the freshwater reservoir effect into account. The faunal assemblage at KA5 is composed of 98.8% domesticated animals and 1.2% wild fauna, and of the domesticated animals, ruminants make up 91.6%, with horses at 6.3%[30].

Krasikovskyi I
(Number of Individuals: 1; Individual Archaeology Code: KRS K.3 N-1)
This burial mound cluster is located in the Volga-Ural region of the forest zone in the Tok River basin. Five kurgans contained five Yamnaya-type graves (EBA) and one Abashevo culture grave

(MLBA). The sample for the present study derives from the Abashevo individual, whose grave also contained a ceramic vessel with traces of repair using bronze staples[37]

Radiocarbon date from the individual sampled in the present study:
KRS K.3 N-1: 2145 to 1897 cal BCE (3632±55 BP, SPb-2224)[37]

Krivyanskyi IX

(Number of Individuals: 2; Individual Archaeology Codes: MBA: K.1 N-30; KRI9 K.5 N-6)
We presented the details of the Krivyanskyi IX cemetery in the EBA section above, as individuals from the site sampled in the current study come from both the EBA and M/LBA. The mound cluster consists of four mounds and 92 burials located between three small tributaries of the Don River; the earliest phase of their building dates to the EBA (No. 1, 2, 4) and MBA (No. 5). The EBA burials were pit graves, and the MBA burials were placed within catacombs. Individual and collective burials (including up to five skeletons) and the use of ochre were characteristic in both periods. Grave goods were rare in all burials, with the exception of a single ceramic vessel in one of the Yamnaya culture burials, as well as a stone flake of small cattle bones in one of the burials of the Catacomb culture. There are no radiocarbon dates for the MBA individuals[19].

Lopatino I

(Number of Individuals: 1) (Individual Archaeology Code: LOP K.3 N-2)
This large kurgan cemetery with 35 burial mounds is located on the Sok River, in the forest-steppe zone on the left bank of the Middle Volga. Eight burial mounds contained pit graves of the Yamnaya (EBA), Poltavka (MBA), and Srubnaya (LBA) cultures. The individual in our study is from the MBA complex, which has not been dated.

Lopatino II

(Number of Individuals: 1) (Individual Archaeology Code: LOP2 K.1 N-1)
This burial mound is located on the Sok River, two km distant from Lopatino I. The kurgan cemetery included four mounds, three of them excavated: one was constructed in the EBA (Yamnaya culture), while two contained graves of the Potapovka tradition. The individual analyzed in the present study dates to the end of the Bronze Age, as confirmed by associated artifacts and a radiocarbon date[22].

Radiocarbon date from the individual sampled in the present study:
LOP2 K.1 N-1: 2887-2636 cal BCE (4180±30 BP, Beta-392492)[22]

Potapovka I

(Number of Individuals: 1; Individual Archaeology Code: POT K.5 N-3)
This kurgan cemetery is located in the Middle Volga region, in the forest-steppe zone, and four of the five mounds that have been excavated together contained 31 burials of two MBA cultural groups: Poltavka (n=6) and Potapovka (n=25). The Potapovka burials are similar to the Sintashta tradition[13] and contain numerous finds and sacrificed domestic animals (horses, cows, sheep, and goats). A series of previously published dates from the cemetery complex confirms its assignment to the MBA[13].

Radiocarbon date from the individual sampled in the present study:
POT K.5 N-3: 2113-1620 cal BCE (3510±80, OxA-4266)[13]

Shumayevo II

(Number of Individuals: 1; Individual Archaeology Code: SHU2 K.6 N-1)

This large burial mound (containing 12 individuals) is located in the Pre-Urals, on the left bank of the Irtek River, a tributary of the Ural River. Material artifacts from all periods of the Bronze Age, as well as the Early Iron Age and the Medieval period, were recovered during excavations. The individual sampled in the present study originated from Burial 1 of kurgan 6, and did not contain any grave goods, but can be clearly assigned to the MBA based on the burial style[38].

Utevka VI

(Number of Individuals: 5; Individual Archaeology Codes: UTE6 K4 N-5; UTE6 K4 N-6; UTE6 K.4 N-1; UTE6 K.6 N-2 K-1; UTE6 K-6 N-6)

This burial ground is located in the Middle Volga region, in the forest-steppe zone. Seven excavated kurgans mostly contained the graves of the Potapovka culture group (similar to the Sintashta tradition). They contained weapons, horse burials, and equipment for chariots, as well as gold ornaments. While most Sintashta sites were fortified settlements, the Potapovka people were more likely mobile pastoralists[39].

Radiocarbon dates from individuals sampled in the present study:
UTE6 K.4 N-1: 2047 to 1767 cal BCE (3583±52 BP, AA-53802)
UTE6 K.6 N-2 K-1: 2468-1925 cal BCE (3760±100 BP, AA-12568)
UTE6 K-6 N-6: 2143 to 1696 cal BCE (3580±80 BP, OxA-4264)

2. Previous lipid study by Mileto et al., 2017

While the present study points to a clear shift in milk consumption patterns between the Eneolithic and EBA in the Pontic-Caspian Steppe, a previous study of ceramic lipid residues from sites located in the steppe zone of Ukraine[40] found some evidence for milk consumption prior to the EBA. Lipids from ruminant dairy products were recovered from ceramics at one site classified as Mid-Eneolithic and three sites considered to be Late Eneolithic/Early Bronze Age. While these findings seem to contradict our results from the Eneolithic Volga steppes, where no dairy proteins were identified for the same time period, the three sites identified as Late Eneolithic/Early Bronze Age sites in the lipid study are in fact normally regarded as EBA Yamnaya[41–45]. While some scholars disagree on the timing of Yamnaya origins with early (*ca*. 3800/3900 BCE[23,24,46]), middle (*ca*. 3500/3300 BCE[1,13,47–49]), and late (*ca*. 3000 BCE[40,50–52]) onsets, using any of these chronologies, ubiquitous dairy consumption only occurs during the tenure of the Yamnaya (Supplementary Table S2). The only evidence for dairy lipids in steppe pottery that precedes the EBA Yamnaya when the more widely accepted middle chronology (or the late chronology) is employed occurs at Mikhailovka I (*ca*. 3800-3000 BCE), which may reflect an initial stage in the production of dairy foods in the steppe, just prior to the rapid spread of the EBA Yamnaya culture[40]. If small-scale dairy use did occur in this period, it could explain the casein peptides found in our single Eneolithic steppe individual. Our results suggest that whatever the date of arrival of dairying, milk was probably not broadly consumed across steppe communities until *ca*. 3300 BCE.

3. Reassignment of period designations from lipid study of Mileto et al., 2017

The time period designations employed in the Mileto et al.[40] study are debated by regional experts. Here, we outline the long, middle and short chronologies that exist for the transition from the Eneolithic into the Early Bronze Age Yamnaya culture, and explain why we support the widely accepted middle chronology that would reassign most of Mileto et al.'s milk findings[40] to the Early Bronze Age (see also Supplementary Table S2).

The Yamnaya chronology has been established for some decades. Initially, in the 1950s, Yamnaya culture sites were designated as Late Neolithic[53]. In the 1960s, however, radiocarbon dates, a growing number of metal artifacts discovered in Yamnaya graves, and typological links with the Maikop culture combined to prompt the widespread acceptance of the Yamnaya culture as dating to the beginning of the EBA. N. I. Merpert's classic synthesis of the Yamnaya culture[42] included two important Yamnaya settlement sites, the Repin site on the Don River and Mikhailovka II on the Dnieper River, as chronological and typological anchors for what was otherwise a chronology based entirely on graves, few of which presented stratified sequences. Repin and layer II at Mikhailovka, which contained Repin-style pottery, are accepted as defining early Yamnaya artifact assemblages and the start of the EBA by prominent experts on the Yamnaya culture in Ukraine and Russia[54–56]. The published dates for Repin and Mikhailovka II (from bone samples) range between 3400-3000 cal BCE. Layer III at Mikhailovka was defined as late Yamnaya by the archaeologists who excavated the site[45]. Rassamakin's proposal to make the EBA and early Yamnaya begin with Mikhailovka layer III[51] – a late chronology for the EBA – therefore, requires complex adjustments. In the Mileto et al.[40] chronology, following Rassamakin[52], Mikhailovka II and other Yamnaya sites like it are re-assigned to the preceding Eneolithic phase. While Rassamakin and Mileto support a late start of the Yamnaya cultural horizon, others, such as Morgunova[23,24,46], maintain an earlier Yamnaya origin (ca. 3800/3900 BCE). Here we use a more conservative and widely accepted "middle" chronology, mid-way between the more occasionally referenced early and late chronologies. When interpreting the Mileto et al.[40] results within the more standard middle chronology, there is only one Late Eneolithic site prior to the onset of the Yamnaya cultural horizon, Mikhailovka level I, that contained possible ruminant dairy lipid signatures. Importantly, no matter which chronology is utilized, and whether the material studied is milk lipids or proteins, it is clear that dairy use did not become widely used until the Yamnaya period (*ca*. 3300 BCE).

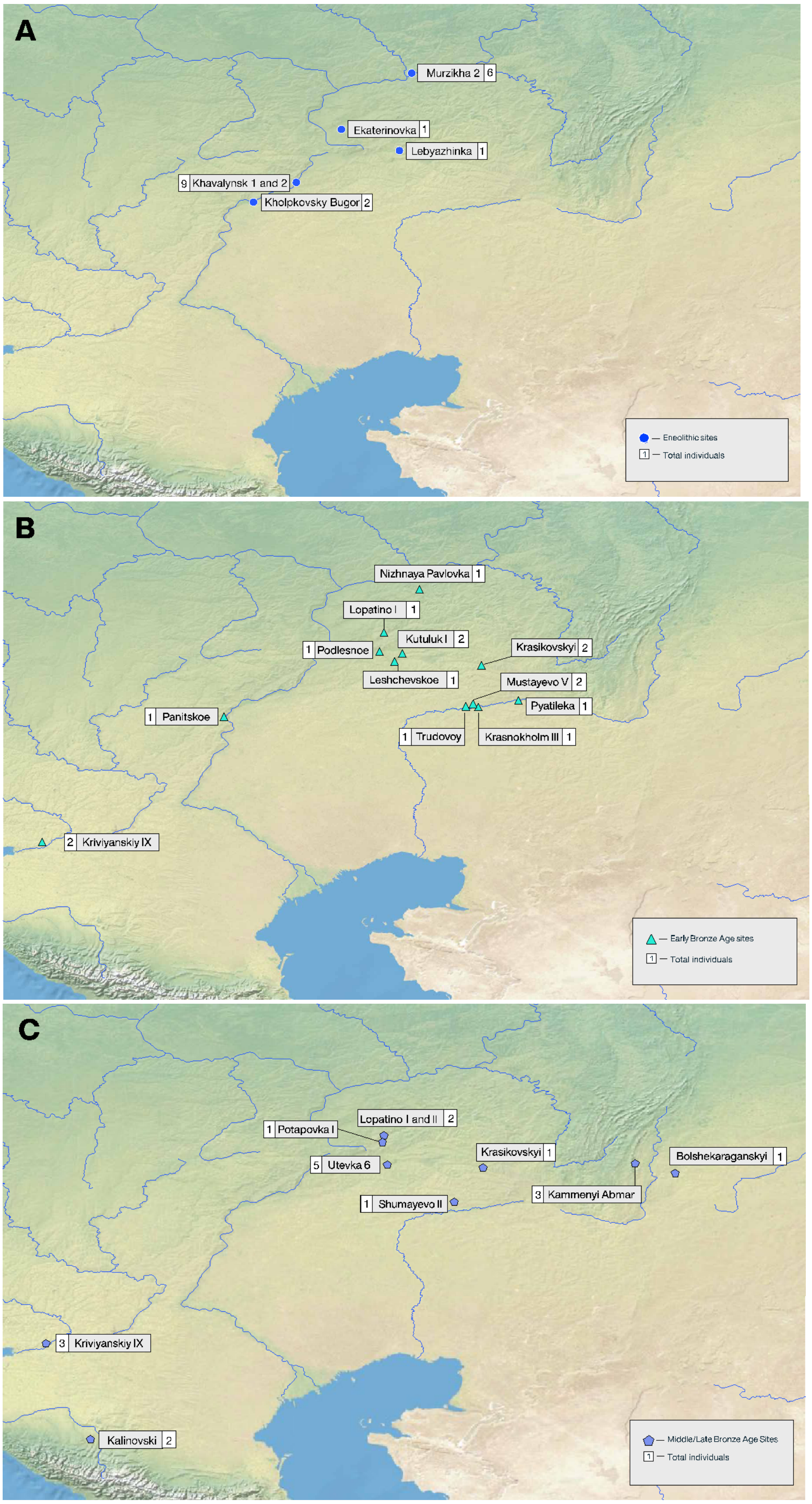

Supplementary Figure S1 – Maps of all sites and individuals included in this study from the (A) Eneolithic; (B) Early Bronze Age; and (C) Middle and Late Bronze Age. Base maps were created

using QGIS 3.12 [1] https://qgis.org/en/site/ and uses Natural Earth vector map data from [2] https://www.naturalearthdata.com/downloads/.

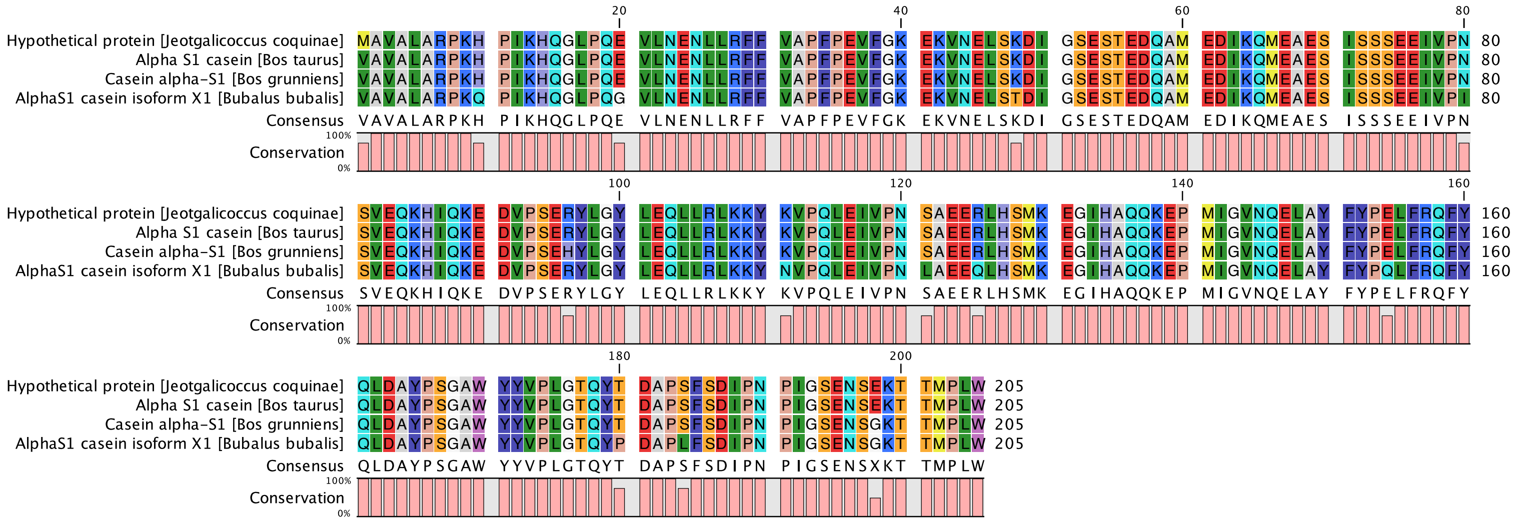

Supplementary Figure S2 – Amino acid alignment comparison of alpha S1 casein from three Bovinae species and *Jeotgalicoccus* showing the small amount of difference between the hypothetical bacterial protein and ruminant casein.

References

1. Schulting, R. J. & Richards, M. P. in *A Bronze Age Landscape in the Russian Steppes: the Samara Valley Project* (eds. Anthony, D. W., Dorcas R. Brown, A. A. K. & Mochalov, O. D.) (books.google.com, 2016).

2. Fernandes, R., Rinne, C., Nadeau, M.-J. & Grootes, P. Towards the use of radiocarbon as a dietary proxy: Establishing a first wide-ranging radiocarbon reservoir effects baseline for Germany. *null* **21,** 285–294 (2016).

3. Fernandes, R., Grootes, P., Nadeau, M.-J. & Nehlich, O. Quantitative diet reconstruction of a Neolithic population using a Bayesian mixing model (FRUITS): The case study of Ostorf (Germany). *Am. J. Phys. Anthropol.* **158,** 325–340 (2015).

4. Korolev, A. I., Kochkina, A. F., Stashenkov, D. A., Khokhlov, A. A. & Roslyakova, N. V. The

Unique Burial of the Ekaterinovsky Cape Early Eneolithic Cemetery in the Middle Volga Region. *Stratum Plus* 285–302 (2018).

5. Korolev, A., Kochkina, A. & Stashenkov, D. The Early Eneolithic burial ground at Ekaterinovsky Cape in the forest-steppe Volga region. *Documenta Praehistorica XLVI* (2019).
6. Anthony, D. W., Khokhlov, A. A., Agapov, S. A., Agapov D. S., Schulting, R., Olalde, I. & Reich, &. D. The Eneolithic cemetery at Khvalynsk on the Volga River. *Praehistorische Zeitschrift* (2021).
7. Malov, N. M. Khlopkovsky burial ground and historiography of the Eneolithic of the Lower Volga region. *Archaeology of the East European forest-steppe* **6,** 32–134 (2008).
8. Kirillova, I. V., Levchenko, V. A., Ippolitov, A. P., Pokrovsky, B. G., Shishlina, N. I. & Yanina, T. A. The origin of objects of invertebrate descent from the Khvalynsk Eneolithic cemeteries (Northern Caspian region). *Quaternary International* **465,** 142–151 (2018).
9. Agapov, S. A. *Khvalynsky Eneolithic Burial Grounds and Khvalynsk Culture*. (Material Research, 2010).
10. Serikov, Y. B. Unusual collective burial of the Neolithic Age in the Kumyshanskaya Cave (Middle Urals). *Bulletin of Archaeology, Anthropology, and Ethnography* **2,** 4–10 (2013).
11. Miller, A. R. V., Ventresca Miller, A. R., Spengler, R., Haruda, A., Miller, B., Wilkin, S., Robinson, S., Roberts, P. & Boivin, N. Ecosystem engineering among ancient pastoralists in northern Central Asia. *Frontiers in Earth Science* **8,** (2020).
12. Anthony, D. W., Brown, D., Brown, E., Goodman, A., Kochlov, A., Kosincev, P. A., Kuznetsov, P., Mochalov, O., Murphy, E. M., Peterson, D. & Others. The Samara valley project: Late bronze age economy and ritual in the Russian steppes. *Eurasia Antiqua* **11,** 395–417 (2005).
13. Anthony, D. W. *The Horse, the Wheel, and Language*. (Princeton University Press, 2007).
14. Hanks, B. Archaeology of the Eurasian steppes and Mongolia. *Annu. Rev. Anthropol.* **39,** 469–486 (2010).
15. Stobbe, A., Gumnior, M., Rühl, L. & Schneider, H. Bronze Age human-landscape interactions in the southern Transural steppe, Russia: evidence from high-resolution palaeobotanical studies. *Holocene* **26,** 1692–1710 (2016).

16. Hollund, H. I., Higham, T., Belinskij, A. & Korenevskij, S. Investigation of palaeodiet in the North Caucasus (South Russia) Bronze Age using stable isotope analysis and AMS dating of human and animal bones. *J. Archaeol. Sci.* **37,** 2971–2983 (2010).
17. Bogdanov, S. V. *The Copper Age of the Steppe Pre-Urals*. (Ural Branch of the Russian Academy of Sciences, 2004).
18. Morgunova, N. L. et al. Krasnikovsky burial ground of the 1st Bronze Age in the Orenburg region. *Arkheologicheskiye pamyatniki Orenburzh'ya* **14,** 4–39 (2019).
19. Bespalyaya, O. I. . *Research report on the mound of 2 objects of archeological heritage of federal significance: 'Krivyansky VII' mound group (2 mounds) " and mounds of 1–7 of the archeological heritage object of federal significance 'Krivyansky IX mound group (3 mounds)' in the construction zone of the greenhouse complex Donskoy in the north-eastern part of the village of Krivyanskaya in the Oktyabrsky district of the Rostov region in 2016*. (Rostov-on-Don, 2016).
20. Peterson, D. L., Northover, P., Salter, C., Maldonado, B. & Anthony, D. W. in *A Bronze Age Landscape in the Russian Steppes: The Samara Valley Project* (ed. Anthony, D. W., Dorcas R. Brown, A. A. K. & Mochalov, O. D.) 291 (Cotsen Institute of Archaeology Press, 2016).
21. Kuznetsov, P. An Indo-Iranian symbol of power in the earliest steppe Kurgans. *Journal of Indo-European Studies* (2005).
22. Vasiliev, I. B., Kuznetsov, P. F., Turesky, M. A. & Semenova, A. P. *The History of the Samara Volga Region from Ancient Times to the Present Day*. (Samara Scientific Center RAS Publishing House, 2000).
23. Morgunova, N. L., Kraeva L A & Matyushko, I. V. Mound burial site Mustaevo V. *Arkheologicheskiye pamyatniki Orenburzh'ya* **7,** 5–49 (2005).
24. Morgunova, N. L. *The Ural Group of Monuments in the System of the Volga-Ural Variant of the Yamnaya Cultural and Historical Group*. (OGPU Publishing House, 2014).
25. Mimokhod, R. A. *Mounds of the Bronze Age - Early Iron Age in the Saratov Volga: characteristics and cultural-chronological attribution of complexes*. (Institute of Archaeology RAS, 2009).

26. Ventresca Miller, A., Usmanova, E., Logvin, V., Kalieva, S., Shevnina, I., Logvin, A., Kolbina, A., Suslov, A., Privat, K., Haas, K. & Rosenmeier, W. Subsistence and social change in central Eurasia: stable isotope analysis of populations spanning the Bronze Age transition. *Journal of Archaeological Science* **42,** 525–538 (2014).

27. Ventresca Miller, A., Usmanova, E., Logvin, V., Kalieva, S., Shevnina, I., Logvin, A., Kolbina, A. & Suslov, A. Dental health, diet, and social transformations in the Bronze Age: Comparative analysis of pastoral populations in northern Kazakhstan. *Quat. Int.* **348,** 130–146 (2014).

28. Chechushkov, I. V. & Epimakhov, A. V. Eurasian steppe chariots and social complexity during the Bronze Age. *Journal of World Prehistory* **31,** 435–483 (2018).

29. Johnson, J. A. & Hanks, B. in *Beyond Elites: Alternatives to Hierarchical Systems in Modelling Social Formations* **215,** 355–367 (Institut für Archäologische Wissenschaften der Universität, 2012).

30. Hanks, B., Miller, A. V., Judd, M., Epimakhov, A., Razhev, D. & Privat, K. Bronze Age diet and economy: New stable isotope data from the Central Eurasian steppes (2100-1700 BC). *Journal of Archaeological Science* **97,** 14–25 (2018).

31. Lindstrom, R. W. Anthropological characteristics of the population of the Bolshekaragansky cemetery, Kurgan 25. *Arkaim: Necropolis: By the Material of Mound* **25,** (2002).

32. Privat, K. in *Arkaim: Necropolis (By the Material of Mound 25 Bolshekaraganskogo Burial: Book* (ed. Zdanovich, D.) (Iuzhno-Ural'skoe Knizhnoe Izdatel'stvo, 2002).

33. Samara Regional Museum of Local Lore. Kalinovsky I burial mound. (2006).

34. Bersenev, A. G., Koryakova, L. N., Chechushkov, I. V. & Sharapova, S. V. Cheek-pieces for a horse harness from the Kamenny Ambar settlement. *Archaeology, Ethnology and Anthropology of Eurasia* **42,** 46–54 (2014).

35. Koryakova, L. N., Krause, R., Epimakhov, A. V., Sharapova, S. V., Panteleyeva, S. E., Berseneva, N. A., Fornasier, J., Kaiser, E., Molchanov, I. V. & Chechushkov, I. V. Archaeological studies of the Kamenny Ambar (Olgino) fortified settlement. *Archaeology, Ethnology and Anthropology of Eurasia* **39,** 61–74 (2011).

36. Ventresca Miller, A., Hanks, B. K., Judd, M., Epimakhov, A. & Razhev, D. Weaning practices

among pastoralists: New evidence of infant feeding patterns from Bronze Age Eurasia. *Am. J. Phys. Anthropol.* **162,** 409–422 (2017).

37. Morgunova, N. L. & Kulkova, M. A. Results of radiocarbon dating of the burial mound Krasikovsky I. *Arkheologicheskiye pamyatniki Orenburzh'ya* 39–45 (2019).
38. Morgunovka, N. L. *Shumayevo mounds*. (Orenburg State Publishing House, 2003).
39. Kuznetsov, P. F. The emergence of Bronze Age chariots in eastern Europe. *Antiquity* **80,** 638–645 (2006).
40. Mileto, S., Kaiser, E., Rassamakin, Y., Whelton, H. & Evershed, R. P. Differing modes of animal exploitation in North-Pontic Eneolithic and Bronze Age Societies. *STAR: Science & Technology of Archaeological Research* **3,** 112–125 (2017).
41. Lagodovskaya, E. F., Shaposhnikova, O. G. & Makarevich, M. L. Osnovnye itogi issledovaniya Mikhailovskogo poseleniya. *Kratkie Soobshcheniya Institut Arkheologii* **9,** 21–28 (1959).
42. Merpert, N. I. *Drevneĭshie skotovody Volzhsko-Ural'skogo mezhdurech'ia*. (Nauka, 1974).
43. Kotova, N. S. & Spitsyna, L. A. in *The Foundations of Radiocarbon Chronology of Cultures between the Vistula and Dnieper: 4000-1000 BC* (eds. Kośko, A. & Klochko, V. I.) 121–131 (Adama Mickiewicza University, Institute of Eastern Studies, Institute of Prehistory, 2003).
44. Korobkova, G. F. & Shaposhnikova, O. G. *Poselenie Mikhailovka—etalonnyi pamiatnik drevneiamnoi kul'tury (Mihailovka settlement—a reference site of the oldest Pit-grave culture)*. (Evropeiskii dom, 2005).
45. Lagodovskaya, E. F., Shaposhnikova, O. G. & Makarevich, M. L. *Mikhailovka Poseleniye*. (Naukova Dumka, 1962).
46. Morgunova, N. L. & Turetskij, M. A. Archaeological and natural scientific studies of Pit-Grave culture barrows in the Volga-Ural interfluve. *Estonian Journal of Archaeology* **20,** 128–149 (2016).
47. Khokhlov, A. A. in *A Bronze Age Landscape in the Russian Steppes: The Samara Valley Project* (ed. Anthony, D. W., Dorcas R. Brown, A. A. K. & Mochalov, O. D.) 105–125 (Cotsen Institute of Archaeology Press, 2016).
48. Anthony, D. W. & Ringe, D. The Indo-European homeland from linguistic and archaeological

perspectives. *Annu. Rev. Linguist.* **1,** 199–219 (2015).

49. Anthony, D. W., Brown, D. R., Khokhlov, A. A., Kuznetsov, P. F. & Mochalov, O. D. *Bronze Age Landscape in the Russian Steppes: The Samara Valley Project*. (Cotsen Institute of Archaeology Press, 2016).
50. Mileto, S., Kaiser, E., Rassamakin, Y. & Evershed, R. P. New insights into the subsistence economy of the Eneolithic Dereivka culture of the Ukrainian North-Pontic region through lipid residues analysis of pottery vessels. *Journal of Archaeological Science: Reports* **13,** 67–74 (2017).
51. Rassamakin, Y. in *Transitions to the Bronze Age: Interregional Interaction and Socio-Cultural Change in the Third Millennium BC Carpathian Basin and Neighbouring Regions* (eds. Heyd, V., Kulcsár, G. & Szeverényi, V.) 113–138 (Archaeolingua, 2013).
52. Rassamakin, Y. in *Late Prehistoric Exploration of the Eurasian Steppe* (eds. Levine, M., Rassamakin, Y., Kislenko, A. & Tatarintseva, N.) 59–182 (McDonald Institute for Archaeological Research Cambridge, 1999).
53. Gimbutas, M. *The prehistory of eastern Europe*. (Peabody Museum, 1956).
54. Telegin, D. Y., Pustovalov, S. Z. & Kovalyukh, N. N. in *The Foundations of Radiocarbon Chronology of Cultures between the Vistula and Dnieper: 4000-1000 BC* (eds. Kośko, A. & Klochko, V. I.) (University Adama Mickiewicza, Institute of Prehistory, 2003).
55. Trifonov, V. A. Repinskaya kultura i protsess slozheniya yamnoy kulturno-istoricheskoy obschnosti (The Repin culture and the process of Yamnaya culture origin). *Antiquities of the Volga-Don steppes in the East Europe Bronze Age system. Volgograd, Peremena* 3–5 (1996).
56. Shishlina, N. & Šišlina, N. I. *Reconstruction of the Bronze Age of the Caspian steppes: Life styles and life ways of pastoral nomads*. (Archaeopress, 2008).
