# Supplementary material for: Dairying enabled Early Bronze Age Yamnaya steppe expansions
Source: Nature. 2021 Sep 15;598(7882):629–33. doi: 10.1038/s41586-021-03798-4 (PMC8550948; doi:10.1038/s41586-021-03798-4)
Supplement: Supplementary file 2 — Reporting Summary [file 41586_2021_3798_MOESM2_ESM.pdf]

nature research

Corresponding author(s): Shevan Wilkin
Nicole Boivin

Last updated by author(s): Jun 14, 2021

Reporting Summary

Nature Research wishes to improve the reproducibility of the work that we publish. This form provides structure for consistency and transparency in reporting. For further information on Nature Research policies, see Authors & Referees and the Editorial Policy Checklist.

Statistics

For all statistical analyses, confirm that the following items are present in the figure legend, table legend, main text, or Methods section.

n/a	Confirmed	
☐	☒	The exact sample size (*n*) for each experimental group/condition, given as a discrete number and unit of measurement
☒	☐	A statement on whether measurements were taken from distinct samples or whether the same sample was measured repeatedly
☒	☐	The statistical test(s) used AND whether they are one- or two-sided<br>*Only common tests should be described solely by name; describe more complex techniques in the Methods section.*
☒	☐	A description of all covariates tested
☒	☐	A description of any assumptions or corrections, such as tests of normality and adjustment for multiple comparisons
☒	☐	A full description of the statistical parameters including central tendency (e.g. means) or other basic estimates (e.g. regression coefficient) AND variation (e.g. standard deviation) or associated estimates of uncertainty (e.g. confidence intervals)
☒	☐	For null hypothesis testing, the test statistic (e.g. F, t, r) with confidence intervals, effect sizes, degrees of freedom and P value noted<br>*Give P values as exact values whenever suitable.*
☒	☐	For Bayesian analysis, information on the choice of priors and Markov chain Monte Carlo settings
☒	☐	For hierarchical and complex designs, identification of the appropriate level for tests and full reporting of outcomes
☒	☐	Estimates of effect sizes (e.g. Cohen's d, Pearson's r), indicating how they were calculated

Our web collection on statistics for biologists contains articles on many of the points above.

Software and code

Policy information about availability of computer code

Data collection	Mass spectrometry analysis was performed on a Q Exactive HF mass spectrometer (Thermo Scientific) equipped with a Digital PicoView source (New Objective) and coupled to a M-Class or nanoAcquity UPLC (Waters).
Data analysis	To transfer raw MS/MS files to Mascot Generic Files (mgf) we used the program MSConvert from the ProteoWizard software package version 3.0.11781. Then, data were searched using Mascot (version 2.6.0) and filtered using MS-MARGE (https://bitbucket.org/rwhagan/ms-marge/src/master/)

For manuscripts utilizing custom algorithms or software that are central to the research but not yet described in published literature, software must be made available to editors/reviewers. We strongly encourage code deposition in a community repository (e.g. GitHub). See the Nature Research guidelines for submitting code & software for further information.

Data

Policy information about availability of data

All manuscripts must include a data availability statement. This statement should provide the following information, where applicable:

- Accession codes, unique identifiers, or web links for publicly available datasets
- A list of figures that have associated raw data
- A description of any restrictions on data availability

All raw, peak, and result protein data has been uploaded to ProteomExchange (http://www.proteomexchange.org). Files are available under the project accession: PXD022300, and the project DOI is 10.6019/PXD022300.

Field-specific reporting

Please select the one below that is the best fit for your research. If you are not sure, read the appropriate sections before making your selection.

☒ Life sciences ☐ Behavioural & social sciences ☐ Ecological, evolutionary & environmental sciences

For a reference copy of the document with all sections, see nature.com/documents/nr-reporting-summary-flat.pdf

Life sciences study design

All studies must disclose on these points even when the disclosure is negative.

Sample size	Sample size was determined by the number of archaeological individuals that had accumulated dental calculus available for collection.
Data exclusions	Dental calculus samples that did not pass the preservation threshold were excluded from further analysis, however, these samples are still listed and their preservation score is provided in Supplementary Table S3.
Replication	These findings can be easily replicated by downloading either the 'raw' or 'mgf' files and re-searching them against the same databases.
Randomization	Samples were not randomized as it was not necessary for the type of study we conducted.
Blinding	As the individuals were not tested for any sort of reaction or non-reaction blinding was not necessary for our study.

Reporting for specific materials, systems and methods

We require information from authors about some types of materials, experimental systems and methods used in many studies. Here, indicate whether each material, system or method listed is relevant to your study. If you are not sure if a list item applies to your research, read the appropriate section before selecting a response.

Materials & experimental systems

n/a	Involved in the study	
☒	☐	Antibodies
☒	☐	Eukaryotic cell lines
☐	☒	Palaeontology
☒	☐	Animals and other organisms
☒	☐	Human research participants
☒	☐	Clinical data

Methods

n/a	Involved in the study	
☒	☐	ChIP-seq
☒	☐	Flow cytometry
☒	☐	MRI-based neuroimaging

Palaeontology

Specimen provenance	Dental calculus samples were collected from Samara State University Department of Archaeology Collection Saplesand the scientific collections of the Museum at the Institute of Plant and Animal Ecology (Ural Branch of the Russian Academy of Sciences).
Specimen deposition	ProteomExchagne http://www.proteomexchange.org/ login: reviewer_pxd022300@ebi.ac.uk and password of: 7TwobDNV
Dating methods	AMS radiocarbon dating was conducted to dates some individuals. Bone sample preparation methods for radiocarbon data follow those as described in Narasimhan et al. Briefly, the outer bone surfaces were removed manually and all samples were soaked in successive washes of methanol, acetone and dichloromethane for 30 min each at room temperature to remove adhesives and consolidants and rinsed in >18.2 MΩ/cm water. Bones were demineralized in 0.5N HCl for 24-36 hr at 5°C, and then gelatinized in 0.01N HCl for 12hr at 60°C. Based on crude gelatin yield and quality, the gelatin was either ultrafiltered (30k Da MWCO), or hydrolyzed for XAD purification. Resulting material was then combusted under vacuum in sealed quartz tubes with CuO and Ag wire, and the resulting CO2 was converted to graphite using H2 reduction over an iron catalyst. Radiocarbon content was measured on a 500kV NEC 1.5SDH-1 compact accelerator, and conventional ages were calculated by normalizing to OXII oxalic acid standards and correcting for fractionation using the δ13C ratio measure on the AMS15.

☒ Tick this box to confirm that the raw and calibrated dates are available in the paper or in Supplementary Information.
